# Supplementary material for: Androgen receptor binding sites enabling genetic prediction of mortality due to prostate cancer in cancer-free subjects
Source: Nat Commun. 2023 Aug 23;14:4863. doi: 10.1038/s41467-023-39858-8 (PMC10447511; doi:10.1038/s41467-023-39858-8)
Supplement: Supplementary file 1 — Supplementary Information [file 41467_2023_39858_MOESM1_ESM.pdf]

Supplementary Information

Androgen receptor binding sites enabling genetic prediction of mortality due to prostate cancer in cancer-free subjects

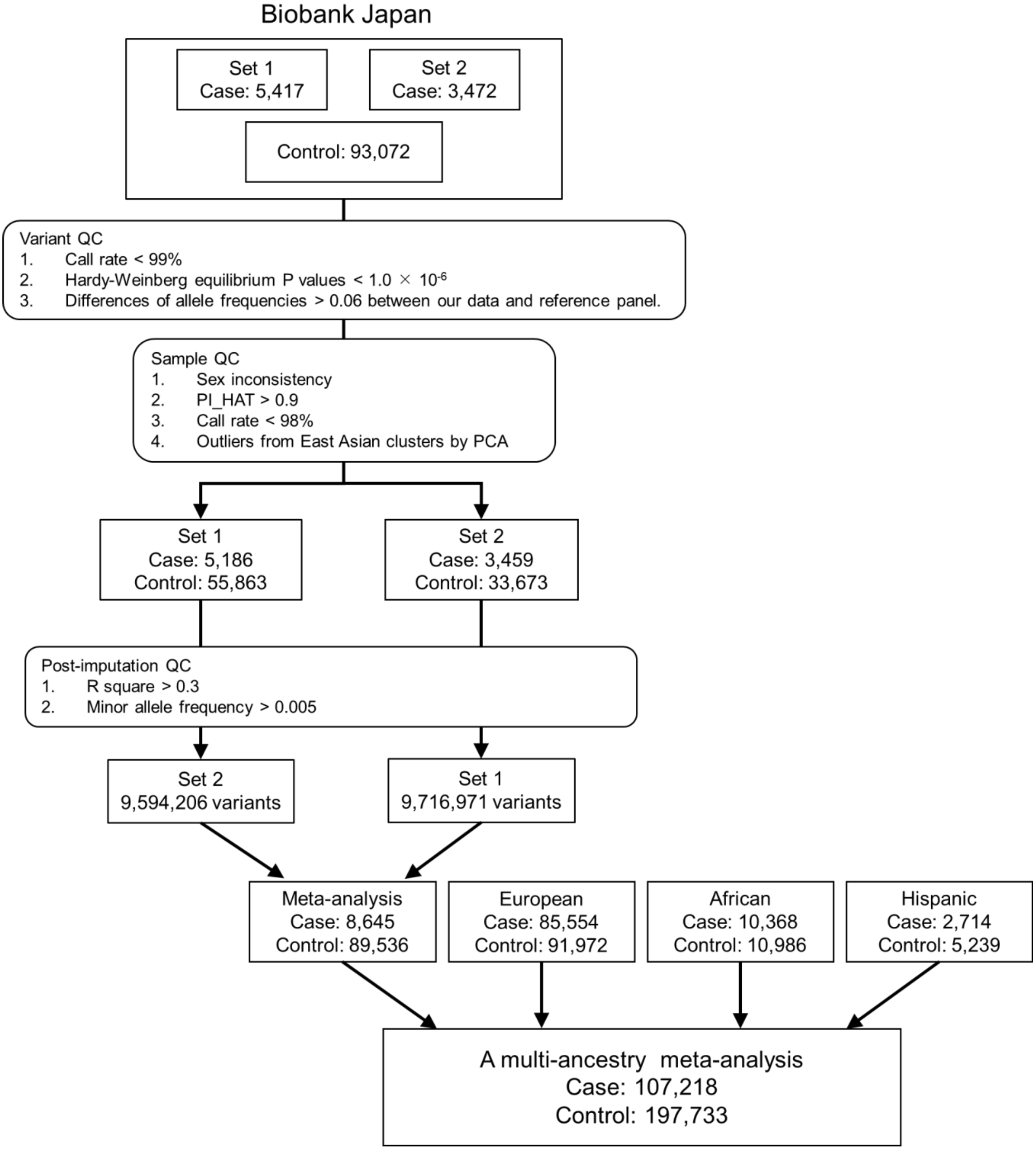

Supplementary Figure 1. An overview of the multi-ancestry meta-analysis for prostate cancer. PCA, principal component analysis; QC, quality control.

a

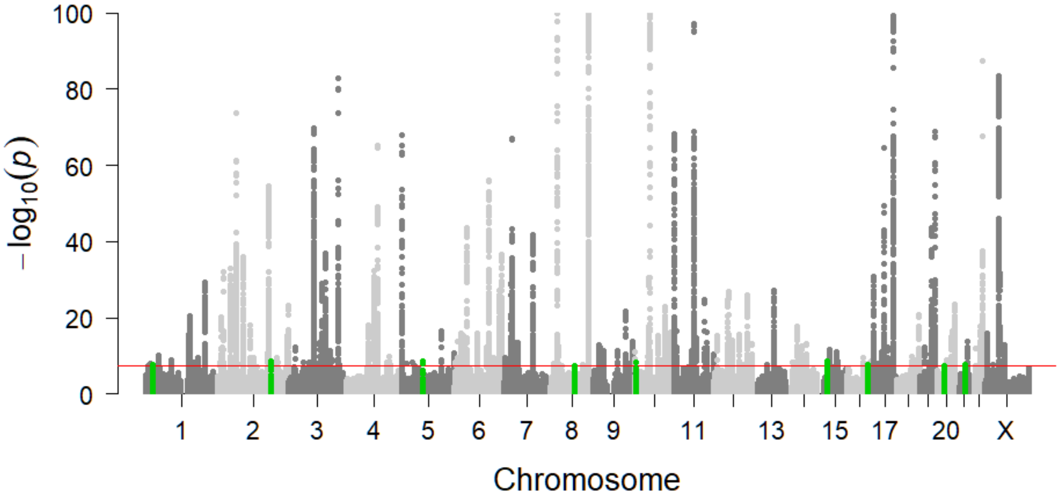

b

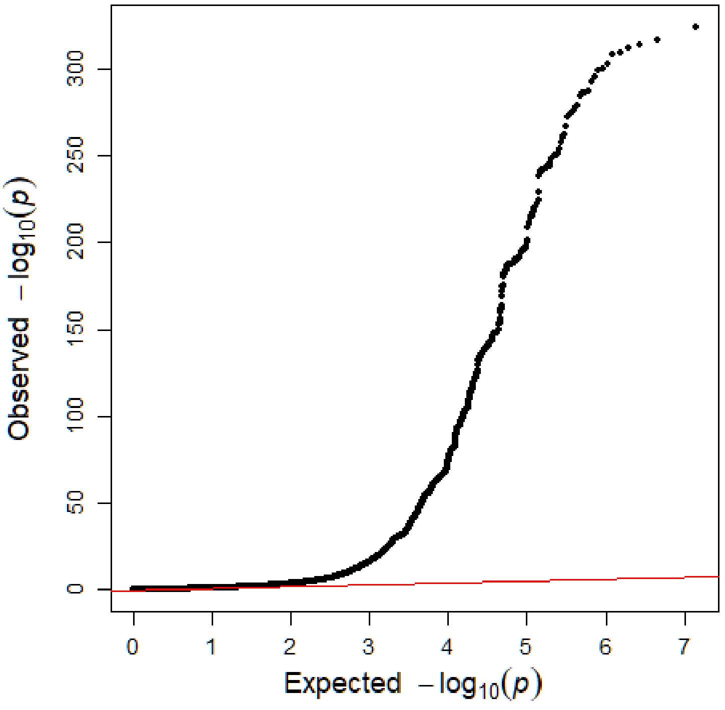

Supplementary Figure 2. The multi-ancestry meta-analysis for prostate cancer.
(a) Manhattan plot. Fixed-effect meta-analysis was performed with METAL. The x-axis is the chromosomal location, and the y-axis is the $-\log_{10}$[p value] for each genetic variant. The horizontal red line represents the genome-wide significance ($p < 5 \times 10^{-8}$). Green points represent novel loci.
(b) Q-Q plot showing the observed versus expected P values.

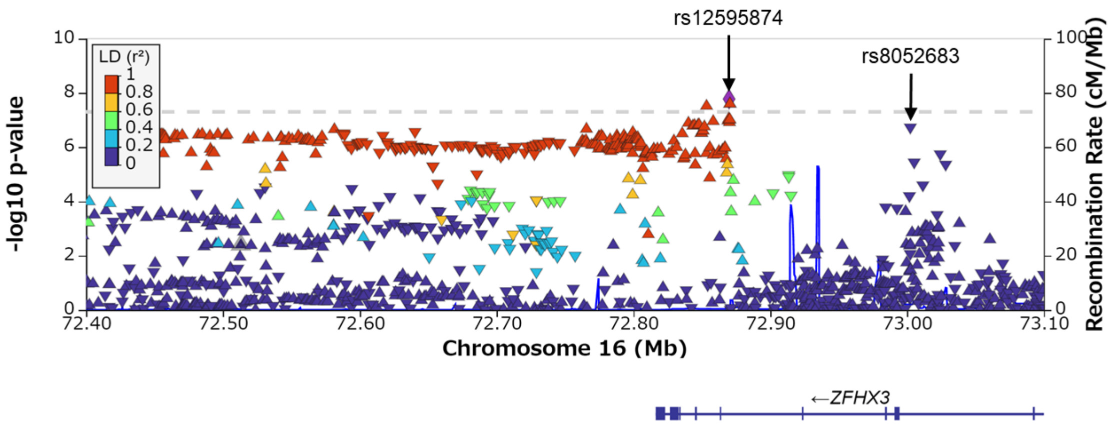

Supplementary Figure 3. A locus plot for the *ZFHX3* region.
While we prioritized rs8052683 in Table 1 based on Bayes factor, this region contains variants exceeding p-value of $5x10^{-8}$ in the fixed effect model.

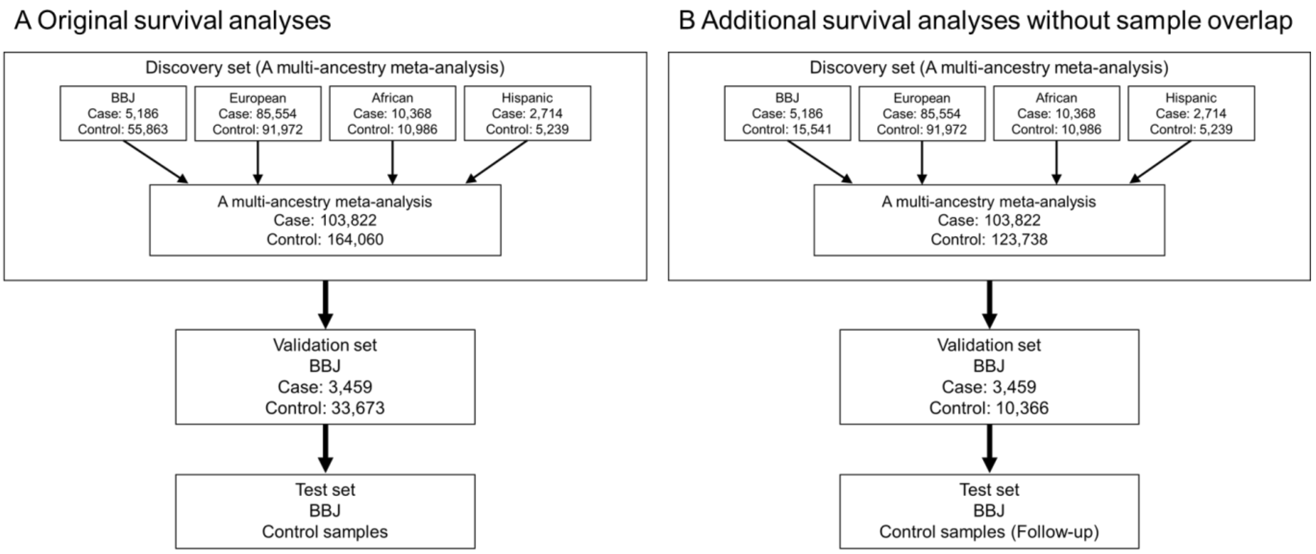

Supplementary Figure 4. A study design for survival analyses.
The data sets used for survival analyses are indicated. We conducted the analyses as shown in the left panel. Then to confirm the findings, we additionally conducted the analyses as shown in the right panel to avoid any sample overlap between survival analyses (test set) and case-control studies.

Supplementary Table 1. Biobank Japan subjects.

Study	Set 1		Set 2	
	Case	Control	Case	Control
**Source**	BBJ 1st cohort	BBJ 1st cohort	BBJ 2nd cohort	BBJ 1st cohort
**Nunmber**	5,186	55,863	3,459	33,673
**male/female**	5,186/0	55,863/0	3459/0	33,673/0
**Age (s.d.)**	72.9 (7.0)	62.8 (13.9)	74.3 (7.3)	62.7 (14.0)
**Genotyping platform**	Illumina OmniExpressExome or combination of Illumina OmniExpress and Illumina HumanExome	Illumina OmniExpressExome or combination of Illumina OmniExpress and Illumina HumanExome	Illumina OmniExpressExome	Illumina OmniExpressExome or combination of Illumina OmniExpress and Illumina HumanExome

The number of samples is after quality control.

BBJ, Biobank Japan

Supplementary Table 2. Genetic correlation between prostate cancer and other diseases in BBJ samples.

Disease	rg	SE	z score	P value	FDR
Peripheral artery disease	-0.300	0.087	-3.410	6.00E-04	0.020
Breast cancer	0.320	0.098	3.280	1.00E-03	0.020
Chronic heart failure	-0.290	0.094	-3.100	1.90E-03	0.025
Cataract	-0.310	0.129	-2.420	0.016	0.153
Cerebral aneurysm	-0.200	0.090	-2.240	0.025	0.172
Ischemic stroke	-0.170	0.077	-2.220	0.026	0.172
Pollinosis	0.240	0.116	2.090	0.036	0.202
Uterine fibroid	0.210	0.120	1.710	0.086	0.392
Stable angina pectoris	-0.130	0.077	-1.690	0.091	0.392
Lung cancer	0.230	0.147	1.600	0.110	0.429
Coronary artery disease	-0.090	0.064	-1.460	0.144	0.511
Graves’ disease	-0.120	0.084	-1.410	0.158	0.512
Colorectal cancer	0.110	0.082	1.320	0.186	0.514
Rheumatoid arthritis	-0.130	0.099	-1.290	0.196	0.514
Nephrotic syndrome	-0.240	0.187	-1.280	0.200	0.514
Arrhythmia	-0.070	0.060	-1.250	0.211	0.514
Urolithiasis	-0.100	0.083	-1.170	0.244	0.557
Chronic hepatitis B	0.160	0.144	1.110	0.265	0.557
Chronic hepatitis C	0.130	0.118	1.070	0.283	0.557
Cervical cancer	0.120	0.118	1.040	0.299	0.557
Pulmonary tuberculosis	0.200	0.189	1.040	0.300	0.557
COPD	-0.110	0.110	-1.000	0.315	0.559
Endometrial cancer	0.150	0.163	0.900	0.369	0.626
Atopic dermatitis	0.070	0.096	0.780	0.438	0.681
Ovarian cancer	0.160	0.226	0.690	0.488	0.681
Cirrhosis	-0.130	0.188	-0.680	0.494	0.681
Unstable angina pectoris	-0.070	0.100	-0.660	0.510	0.681
Hepatocellular carcinoma	0.140	0.216	0.630	0.528	0.681
Keloid	-0.060	0.099	-0.630	0.529	0.681
Pancreatic cancer	0.140	0.236	0.590	0.553	0.681
Myocardial infarction	-0.040	0.072	-0.590	0.558	0.681
Gastric cancer	0.060	0.097	0.580	0.559	0.681
Glaucoma	0.050	0.089	0.530	0.596	0.705
Esophageal cancer	-0.040	0.091	-0.490	0.626	0.718

Drug eruption	-0.200	0.449	-0.450	0.653	0.728
Epilepsy	-0.070	0.221	-0.310	0.754	0.817
Type 2 diabetes	-0.010	0.056	-0.220	0.825	0.848
Osteoporosis	-0.020	0.101	-0.220	0.826	0.848
Asthma	-0.010	0.073	-0.180	0.861	0.861

BBJ, Biobank Japan; SE, standard error; FDR, false discovery rate

Supplementary Table 3. Genetic correlation between prostate cancer and other diseases in Europeans.

Disease	rg	SE	z score	P value
Peripheral artery disease	-0.072	0.053	-1.326	1.85E-01
Breast cancer	0.112	0.041	2.714	6.70E-03
Chronic heart failure	-0.055	0.039	-1.403	1.61E-01

SE, standard error

Supplementary Table 4. Enrichment analysis for active enhancer by cell groups.

Cell group	Enrichment	P value	FDR
Gastrointestinal	4.301	5.60E-05	0.001
Cardiovascular	4.273	4.14E-03	0.017
Hematopoietic	2.349	5.18E-03	0.017
Adrenal pancreas	5.178	0.014	0.035
Other	2.428	0.018	0.036
Connective bone	2.602	0.033	0.047
Liver	3.923	0.029	0.047
Kidney	4.705	0.061	0.077
Skeletal muscle	2.718	0.092	0.102
Central nerve system	1.665	0.319	0.319

FDR, false discovery rate

Supplementary Table 5. Exonic variants with posterior probability > 0.1.

rsID	Chr	Position	Ref	Alt	Gene	Annotation	META			BBJ		EUR		AFR		HIS	
							Freq	PP	P-value	Freq	P-value	EUR	P-value	AFR	P-value	Freq	P-value
rs1078004	2	85769711	C	G	*MAT2A*	synonymous	0.445	0.10	3.29E-36	0.364	1.24E-04	0.463	8.63E-28	0.679	1.73E-05	0.400	2.35E-02
rs11900747	2	121103903	T	G	*INHBB*	missense	0.150	0.40	3.54E-10	0.233	3.96E-03	0.078	5.01E-07	0.406	1.32E-02	0.091	3.21E-01
rs76832527	2	242157241	G	A	*ANO7*	missense	0.143	0.90	5.25E-24	0.108	1.46E-01	0.173	5.00E-22	0.042	1.05E-02	0.152	3.10E-02
rs2277283	11	61908440	T	C	*INCENP*	missense	0.226	1.00	2.83E-12	0.080	3.32E-01	0.316	1.61E-10	0.166	5.24E-02	0.194	3.29E-02
rs2066827	12	12871099	T	G	*CDKN1B*	missense	0.204	1.00	8.94E-10	0.027	4.51E-01	0.242	4.42E-09	0.706	4.55E-02	0.203	8.33E-01
rs138708	22	39138332	G	A	*SUN2*	missense	0.070	0.99	1.96E-18	0.162	4.02E-13	0.020	2.62E-06	0.010	3.87E-01	0.016	1.05E-01

Ref, reference allele; Alt, alternative allele; Freq, allele frequency of alternative allele; PP, posterior probability
META, meta-analysis; BBJ, Biobank Japan; EUR, Europeans; AFR, Africans; HIS, Hispanic

Supplementary Table 6. Enrichment analysis for androgen receptor binding sites by linkage disequilibrium score regression.

Subject	Cell type*	Proportion of variants	Proportion of heritability	Enrichment	P value
BBJ	prostate cancer	0.013	0.376	29.335	3.21E-04
	prostate	0.014	0.459	33.962	6.08E-05
European	prostate cancer	0.012	0.339	27.867	2.37E-05
	prostate	0.013	0.466	36.199	3.94E-09

*from ChIP-atlas.

Supplementary Table 7. GREGOR of androgen receptor binding sites by linkage disequilibrium of European ancestries.

Cell type*	SNP set	Number of SNPs	Number of InBed SNPs	Expect	Fold enrichment	P value
Prostate cancer cells	lead SNPs	166	63	29.704	2.121	1.74E-12
	PP0.1	331	139	59.486	2.337	5.40E-31
	PP0.5	96	31	13.128	2.361	1.25E-07
Prostate cells	lead SNPs	166	82	30.824	2.660	4.52E-26
	PP0.1	331	170	61.720	2.754	4.45E-53
	PP0.5	96	42	13.571	3.095	1.25E-15

GREGOR, Genomic Regulatory Elements and Gwas Overlap algoRithm; SNP, single nucleotide polymorphism
*from ChIP-atlas.

Supplementary Table 8. GREGOR of AR binding sites by LD of Asian ancestries.

Cell type	SNP set	Number of SNPs	Number of InBed SNPs	Expect	Fold enrichment	P value
Prostate cancer cells	lead SNPs	166	64	31.45	2.035	1.55E-11
	PP0.1	331	141	65.22	2.162	2.36E-27
	PP0.5	96	32	14.31	2.236	4.06E-07
Prostate cells	lead SNPs	166	70	32.35	2.164	1.17E-14
	PP0.1	331	146	66.31	2.202	1.23E-29
	PP0.5	96	36	14.59	2.468	1.77E-09

GREGOR, Genomic Regulatory Elements and Gwas Overlap algoRithm; LD, linkage disequilibrium; SNP, single nucleotide polymorphism
PrCa cells, AR binding sites in PrCa cells from ChIP-atlas; Prostate, AR binding sites in Prostate cells from ChIP-atlas.

Supplementary Table 9. Variants with PP > 0.1 located on androgen receptor binding sites.

rsID	Chromosome	Position	Posterior probability
rs1574259	2	173313453	0.12
rs74001374	2	238411293	0.12
rs7591218	2	43637998	1.00
rs10188360	2	8597823	0.25
rs10190752	2	8597888	0.63
rs1283108	3	106961285	0.18
rs78416326	3	170074517	1.00
rs9815122	3	23155056	0.16
rs9872872	3	23155128	0.20
rs2280283	4	152020777	0.10
rs6853490	4	95544718	1.00
rs199577062	5	1889346	1.00
rs1887414	6	109292736	0.19
rs2018336	6	11217897	1.00
rs13215045	6	153447516	0.95
rs150765082	6	160581543	1.00
rs1983891	6	41536427	0.16
rs9472119	6	43709703	0.26
rs9472120	6	43709785	0.38
rs9472121	6	43710015	0.31
rs9655205	7	20999211	0.15
rs67152137	7	27975919	0.23
rs10486567	7	27976563	0.55
rs6465657	7	97816327	0.35
rs6465658	7	97816638	0.11
rs11986220	8	128531689	1.00
rs1160267	8	23529521	0.99
rs12634	9	132573536	0.30

rs17694493	9	22041998	1.00
rs1417078	9	82104658	0.18
rs4962419	10	126697114	0.16
rs12769019	10	126697327	0.16
rs12769682	10	126697494	0.27
rs10993994	10	51549496	1.00
rs75184941	11	134277750	0.12
rs767127	14	69134264	0.86
rs3893264	16	54683802	0.95
rs78012083	16	72870564	0.20
rs78971197	16	72870587	0.20
rs72811264	17	12576704	0.43
rs684232	17	618965	0.98
rs2659124	19	51354597	1.00
rs11698745	20	25216113	0.12
rs5759167	22	43500212	1.00

Supplementary Table 10. Cox proportional hazard model for death from prostate cancer using polygenic risk scores (PRSs).

	score	HR (95%CI)	Pr(>|z|)
GWAS_PRS	quantitative	1.55 (1.32-1.83)	1.6×10^{-7}
	Top 10% vs bottom 50%	3.52 (1.97-6.28)	2.0×10^{-5}
AR_prioritized_PRS	quantitative	1.61 (1.39-1.86)	2.2×10^{-10}
	Top 10% vs bottom 50%	5.57 (3.25-9.54)	4.2×10^{-10}

HR: hazard ratio, CI: confidence interval, GWAS_PRS: PRS simply constructed by GWAS results using P/T method

Supplementary Table 11. Cox proportional hazard model for death from prostate cancer using polygenic risk scores (PRSs) without any sample overlap between case-control and survival studies.

	score	HR (95%CI)	Pr(>|z|)
GWAS_PRS	quantitative	1.44 (1.24-1.67)	$1.7x10^{-7}$
	Top 10% vs bottom 50%	4.01 (2.40-6.70)	$1.1x10^{-5}$
	Top 1% vs bottom 50%	6.61 (2.30-19.04)	$4.6x10^{-4}$
AR_prioritized_PRS	quantitative	2.08 (1.62-2.67)	$9.3x10^{-9}$
	Top 10% vs bottom 50%	4.58 (2.66-7.89)	$4.0x10^{-8}$
	Top 1% vs bottom 50%	16.70 (2.66-7.89)	$8.3x10^{-12}$

HR: hazard ratio, CI: confidence interval, GWAS_PRS: PRS simply constructed by GWAS results using P/T method

Supplementary Table 12. Cox proportional hazard model for death in subjects with prostate cancer using AR-prioritized polygenic risk scores (PRSs).

score	HR (95%CI)	Pr(>|z|)
Top 10% vs bottom 50%	1.08 (0.89-1.30)	0.45
Top 1% vs bottom 50%	1.37 (0.81-2.33)	0.25

HR: hazard ratio, CI: confidence interval

The BioBank Japan Project Consortium

Akihide Masumoto[a], Akiko Nagai[c], Daisuke Obata[c], Hiroki Yamaguchi[d], Kaori Muto[e], Kazuhisa Takahashi[f], Ken Yamaji[g], Kozo Yoshimori[h], Masahiko Higashiyama[i], Nobuaki Sinozaki[j], Satoshi Asai[k], Satoshi Nagayama[l], Shigeo Murayama[m], Shiro Minami[n], Takao Suzuki[o], Takayuki Morisaki[p], Wataru Obara[q], Yasuo Takahashi[r], Yoichi Furukawa[s], Yoshinori Murakami[t], Yuji Yamanashi[u], Yukihiro Koretsune[v]

[a]IIZUKA HOSPITAL, Fukuoka, Japan; [b]Department of Public Policy, Institute of Medical Science, The University of Tokyo, Tokyo, Japan; [c]Center for Clinical Research and Advanced Medicine, Shiga University of Medical Science, Shiga, Japan; [d]Departmentof Hematology, Nippon Medical School, Tokyo, Japan; [e]Department of Public Policy, Institute of Medical Science, The University of Tokyo, Tokyo, Japan; [f]Department of Respiratory Medicine, Juntendo University Graduate School of Medicine, Tokyo, Japan; [g]Department of Internal Medicine and Rheumatology, Juntendo University Graduate School of Medicine, Tokyo, Japan; [h]Fukujuji Hospital, Japan Anti-Tuberculosis Association, Tokyo, Japan; [i]Department of General Thoracic Surgery, Osaka International Cancer Institute, Osaka, Japan; [j]Tokushukai Group, Tokyo, Japan; [k]Division of Pharmacology, Department of Biomedical Science, Nihon University School of Medicine, Tokyo, Japan. Division of Genomic Epidemiology and Clinical Trials, Clinical Trials Research Center, Nihon University. School of Medicine, Tokyo, Japan; [l]The Cancer Institute Hospital of the Japanese Foundation for Cancer Research, Tokyo, Japan; [m]Tokyo Metropolitan Geriatric Hospital and Institute of Gerontology, Tokyo, Japan; [n]Department of Bioregulation, Nippon Medical School, Kawasaki, Japan; [o]Tokushukai Group, Tokyo, Japan; [p]Division of Molecular Pathology IMSUT Hospital, Department of Internal Medicine Project Division of Genomic Medicine and Disease Prevention The Institute of Medical Science The University of Tokyo, Tokyo, Japan; [q]Department of Urology, Iwate Medical University, Iwate, Japan; [r]Division of Genomic Epidemiology and Clinical Trials, Clinical Trials Research Center, Nihon University School of Medicine, Tokyo, Japan; [s]Division of Clinical Genome Research, Institute of Medical Science, The University of Tokyo, Tokyo, Japan; [t]Department of Cancer Biology, Institute of Medical Science, The University of Tokyo, Tokyo, Japan; [u]Division of Genetics, The Institute of Medical Science, The University of Tokyo, Tokyo, Japan; [v]National Hospital Organization Osaka National Hospital, Osaka, Japan
