# Supplementary material for: Androgen receptor binding sites enabling genetic prediction of mortality due to prostate cancer in cancer-free subjects
Source: Nat Commun. 2023 Aug 23;14:4863. doi: 10.1038/s41467-023-39858-8 (PMC10447511; doi:10.1038/s41467-023-39858-8)
Supplement: Supplementary file 3 — Description of Additional Supplementary Files [file 41467_2023_39858_MOESM3_ESM.docx]

**Description of Additional Supplementary Files**

Supplementary Data 1:

Description: 166 credible sets provided by fine-mapping for all significant loci identified by the trans-ethnic meta-analysis. We calculated asymptotic Bayes factor and built a credible set of putative causal variants with an incremental 95% probability.
