# Supplementary material for: Androgen receptor binding sites enabling genetic prediction of mortality due to prostate cancer in cancer-free subjects
Source: Nat Commun. 2023 Aug 23;14:4863. doi: 10.1038/s41467-023-39858-8 (PMC10447511; doi:10.1038/s41467-023-39858-8)
Supplement: Supplementary file 5 — Reporting Summary [file 41467_2023_39858_MOESM5_ESM.pdf]

nature portfolio

Corresponding author(s): Chikashi Terao

Last updated by author(s): Apr 12, 2023

Reporting Summary

Nature Portfolio wishes to improve the reproducibility of the work that we publish. This form provides structure for consistency and transparency in reporting. For further information on Nature Portfolio policies, see our Editorial Policies and the Editorial Policy Checklist.

Statistics

For all statistical analyses, confirm that the following items are present in the figure legend, table legend, main text, or Methods section.

n/a	Confirmed	
☐	☒	The exact sample size (*n*) for each experimental group/condition, given as a discrete number and unit of measurement
☒	☐	A statement on whether measurements were taken from distinct samples or whether the same sample was measured repeatedly
☐	☒	The statistical test(s) used AND whether they are one- or two-sided<br>*Only common tests should be described solely by name; describe more complex techniques in the Methods section.*
☐	☒	A description of all covariates tested
☐	☒	A description of any assumptions or corrections, such as tests of normality and adjustment for multiple comparisons
☐	☒	A full description of the statistical parameters including central tendency (e.g. means) or other basic estimates (e.g. regression coefficient) AND variation (e.g. standard deviation) or associated estimates of uncertainty (e.g. confidence intervals)
☐	☒	For null hypothesis testing, the test statistic (e.g. *F*, *t*, *r*) with confidence intervals, effect sizes, degrees of freedom and *P* value noted<br>*Give P values as exact values whenever suitable.*
☐	☒	For Bayesian analysis, information on the choice of priors and Markov chain Monte Carlo settings
☒	☐	For hierarchical and complex designs, identification of the appropriate level for tests and full reporting of outcomes
☒	☐	Estimates of effect sizes (e.g. Cohen's *d*, Pearson's *r*), indicating how they were calculated

Our web collection on statistics for biologists contains articles on many of the points above.

Software and code

Policy information about availability of computer code

Data collection	All cases and controls in the BBJ GWAS were collected in the BioBank Japan (https://biobankjp.org/english/index.html), which is a biobank that collaboratively collects DNA and serum samples from 12 medical institutions in Japan and recruited approximately 200,000 patients with a diagnosis of at least one of 47 diseases. We used summary statistics from the previous Trans-ancestry GWAS meta analysis of PrCa (PMID: 33398198). We obtained the bed file containing androgen receptor (AR) binding sites from the ChIP-atlas (https://chip-atlas.org/).
Data analysis	We conducted quality control (QC) with PLINK v1.90 and imputation with minimac4; We conducted a GWAS of BBJ samples by applying a generalized linear mixed model using SAIGE (version 0.35.8.3). The Meta-analysis was done with METAL (version March 2011). We conducted statistical fine-mapping with the script available from the following site (https://github.com/chr1swallace/finemap-psa/blob/master/bf-functions.R). The polygenic risk score (PRS) analysis was performed with PLINK v1.9. The survival analysis was conducted with the R package survival (version 3.1-8) (https://cran.r-project.org/web/packages/survival/index.html). We analyzed the heritability enrichment of the GWAS signals of AR binding sites using two methods: LDSC (version 1.0.0) and GREGOR, a SNP-matching-based method to test for enrichment (version 1.4.0 https://genome.sph.umich.edu/wiki/GREGOR). We analyzed genetic correlation between ancestries with Popcorn (version 0.9.9). Plots were provided with R (version 4.0.2).

For manuscripts utilizing custom algorithms or software that are central to the research but not yet described in published literature, software must be made available to editors and reviewers. We strongly encourage code deposition in a community repository (e.g. GitHub). See the Nature Portfolio guidelines for submitting code & software for further information.

Data

Policy information about availability of data

All manuscripts must include a data availability statement. This statement should provide the following information, where applicable:

- Accession codes, unique identifiers, or web links for publicly available datasets
- A description of any restrictions on data availability
- For clinical datasets or third party data, please ensure that the statement adheres to our policy

The GWAS and Meta-analysis summary statistics will be available at http://jenger.riken.jp/result

Human research participants

Policy information about studies involving human research participants and Sex and Gender in Research.

Reporting on sex and gender	Since the target disease is prostate cancer, only male subjects were recruited and analyzed.
Population characteristics	The individuals included in the PrCa GWAS were all Japanese who were collected in the BioBank Japan (https://biobankjp.org/english/index.html), which is a biobank that collaboratively collects DNA and serum samples from 12 medical institutions in Japan and recruited approximately 200,000 patients with a diagnosis of at least one of 47 diseases (including 13 cancers). For meta-analysis, we used summary statistics of PrCa GWAS divided into three ancestries (European, African and Hispanic).
Recruitment	From Biobank Japan (BBJ), we selected 8,645 pathologically proven PrCa patients and 89,536 male subjects without PrCa as control. They were recruited in BioBank Japan as shown above so they are hospital-based participants. The advantage of this study is availability of follow-up data of the participants.
Ethics oversight	The study was approved by the Ethics Committee of RIKEN (17-17-16(16)) and Institute of Medical Sciences at The University of Tokyo.

Note that full information on the approval of the study protocol must also be provided in the manuscript.

Field-specific reporting

Please select the one below that is the best fit for your research. If you are not sure, read the appropriate sections before making your selection.

☒ Life sciences ☐ Behavioural & social sciences ☐ Ecological, evolutionary & environmental sciences

For a reference copy of the document with all sections, see nature.com/documents/nr-reporting-summary-flat.pdf

Life sciences study design

All studies must disclose on these points even when the disclosure is negative.

Sample size	First, we conducted a GWAS of BBJ samples consisting of 8,645 cases and 89,536 controls. We used all available samples we have. samples Next, we conducted a trans-ethnic meta-analysis, using the results of BBJ and the summary statistics of a previous GWAS for prostate cancer including European, African, and Hispanic ancestries assuming a random effect. The combined dataset consisted of 107,218 cases and 197,733 controls.
Data exclusions	For quality control of samples, we excluded those with: (1) a sample call rate of <0.98; and (2) outliers from East Asian clusters identified by a principal component analysis using genotyped samples. For quality control of genotypes, we excluded variants meeting any of the following criteria: (1) call rate < 99%; (2) P value for Hardy-Weinberg equilibrium (HWE) < 1.0 × 10-6. We also excluded SNP with a large allele frequency difference between the reference panel and the samples (> 0.06).
Replication	We conducted trans-ethnic meta-analysis.
Randomization	This study was not applicable because it was not a prospective study.
Blinding	All individual data was anonymized to protect privacy.

Reporting for specific materials, systems and methods

We require information from authors about some types of materials, experimental systems and methods used in many studies. Here, indicate whether each material, system or method listed is relevant to your study. If you are not sure if a list item applies to your research, read the appropriate section before selecting a response.

Materials & experimental systems

n/a	Involved in the study
☒	☐ Antibodies
☒	☐ Eukaryotic cell lines
☒	☐ Palaeontology and archaeology
☒	☐ Animals and other organisms
☒	☐ Clinical data
☒	☐ Dual use research of concern

Methods

n/a	Involved in the study
☒	☐ ChIP-seq
☒	☐ Flow cytometry
☒	☐ MRI-based neuroimaging
